# Impaired T Cell-dependent Humoral Immune Response Associated with Juvenile-onset Recurrent Respiratory Papillomatosis Progression

**DOI:** 10.1038/srep36378

**Published:** 2016-11-08

**Authors:** Xunyao Wu, Guoliang Wang, Xi Chen, Jie Zhang, Jing Zhao, Jun Wang, Yang Xiao, Jun Tai, Shengcai Wang, Guixiang Wang, Hua Wang, Lina Bai, Jingang Gui, Xin Ni

**Affiliations:** 1 Key Laboratory of Major Diseases in Children, Ministry of Education, Beijing Children’s Hospital, Capital Medical University, Beijing, China; 2 Laboratory of Immunology, Beijing Pediatric Research Institute, Beijing Children’s Hospital, Capital Medical University, Beijing, China; 3 Department of Otolaryngology, Head and Neck Surgery, Beijing Children’s Hospital, Capital Medical University, Beijing, China; 4 Department of Otolaryngology Head and Neck Surgery, Beijing Tongren Hospital, Capital Medical University, Beijing, China; 5 Beijing Key Laboratory for Pediatric Diseases of Otolaryngology, Head and Neck Surgery, Beijing Pediatric Research Institute, Beijing Children’s Hospital, Capital Medical University, Beijing, China

## Abstract

Whether humoral immunity plays a role in HPV type 6 or 11 virus-mediated Juvenile-onset Recurrent Respiratory Papillomatosis (JORRP) remains unknown. In the present study, serum total IgG level in 44 JORRP patients was significantly decreased compared with that in 40 healthy controls. Moreover, expanded CD3^−^CD19^+^ B cells with down-regulation of CD23, CD40, HLA-DR and up-regulation of CD86 expression were found in the peripheral blood of JORRP patients. Flow cytometry analysis of B-cell compartment showed that the frequency of both CD19^+^CD27^hi^ plasma cells and CD19^+^CD27^+^ memory B cells were decreased in JORRP patients. Importantly, although the proportion of circulating CXCR5^+^PD1^hi^ Tfh cells was not changed, the function of Tfh cells were greatly impaired with reduced ability of IL-21 secretion to promote B cell maturation. Association analysis by the Kaplan-Meier method revealed that IL-21 secreting Tfh cell was positively correlated to the CD27^+^ B cell subset frequency, the serum IgG level and the frequency of recurrence in JORRP patients, but negatively correlated to the percentage of IgD^+^CD27^−^ B cell. We concluded that a reduced IL-21 secretion by Tfh cells may limit B cell maturation and antibody production in JORRP patients and Tfh cell-derived IL-21 might be associated with JORRP outcome in clinic.

Juvenile-onset Recurrent Respiratory Papillomatosis (JORRP), characterized by recurrent growth of papillomas in the respiratory tract, is the most common benign neoplasm of the larynx in children^1^. Although relatively rare with 1.4 RRP patients per 100,000 in the general UK population and children of about 4.3 per 100,000 in the USA^2^,^3^, JORRP could cause heavy economic burden on a family by multiple surgery procedures to prevent airway obstruction^4^.

While persistent Human Papillomatosis Virus (HPV) type 6 or 11 virus infection is often associated with JORRP development^5^, emerging evidences showed that immune responses against HPV type 6 or 11 virus infection are the determinants of JORRP outcome^6^. It has been suggested that impaired cellular immune response in patients with JORRP support sustained HPV-6/11 infection and prevent HPV virus from clearance. Defective migration of matured DC was shown to associate with severe course of disease and skewed Th1/Th2 T-cell ratio was found in patients with JORRP^7^,^8^.

Virus-specific antibody, plasma cells as well as memory B cells are the main components of long-term humoral immunity in virus-infected individuals and loss of humoral immune balance significantly contributes to cancer development^9^. Meanwhile, T-cell subsets particular T follicular helper (Tfh) cells, have been demonstrated a critical role in orchestrating B-cell-related humoral response via supporting memory B-cell and plasma cell generation, as well as promoting protective antibody production during germinal center (GC) formation^10^,^11^,^12^. Current documentation is lacking on the role of humoral immune response against HPV-6/11 virus infection in JORRP patients. In the present study, patients diagnosed of JORRP were analyzed for serum antibody production, the frequency and subset of B cells in the peripheral blood. We observed a reduction in plasma and memory B cells that was associated with decreased serum IgG production in JORRP patients. In addition, an impaired secretion of IL-21 by Tfh cells, possibly leading to the immaturity of B-cell development, was correlated to a reduced serum IgG level and an increased recurrent frequency in JORRP patient. These results suggest that Tfh-cell-mediated humoral immunity play an important role in the outcome of JORRP in clinic.

## Materials and Methods

### Study Subjects and Ethics

Blood Samples were obtained from 44 patients diagnosed of Juvenile-onset recurrent respiratory papillomatosis and 40 age- and sex-matched healthy donors (p > 0.05) under physical examination for the entrance to the kindergarten or primary school from both Beijing Children’s Hospital and Beijing TongRen Hospital. The ethics was approved by Beijing Children’ Hospitals’ ethics committees (Grant No. 2014-27) and carried out in accordance with approved guidelines. Written informed consents were obtained from all the participants or their parent or legal guardian. The basic physical and clinical information of patients are present in Table 1.

### Peripheral Blood Mononuclear Cells (PBMCs) Isolation

Freshly isolated EDTA anticoagulated blood was diluted with PBS solution and layered carefully on Ficoll-Hypaque density gradients. After centrifuged at 1000 g for 20 minutes at room temperature, interphase cell layer was carefully transferred into new 15 ml tubes. Fill the 15 ml tube with 10 ml PBS, centrifuged at 300 g for 15 minutes. Discard the supernatant completely and viability of isolated PBMCs was determined by trypan blue exclusion staining.

### Flow Cytometry

Human FITC-conjugated anti-IgD and anti-CD86, Alexa Fluo 488-conjugated anti-CXCR5, PE-conjugated anti-CD80 and anti-CD40, PerCP-Cy5.5-conjugated anti-CD23 and anti-CD3ε, APC-conjugated anti-HLA-DR and anti-CD19 were purchased from Biolegend (San Diego, CA, USA). PE-conjugated anti-CD27 and anti-ICOS were purchased from BD Pharmingen (San Diego, CA, USA). Alexa-647-conjugated anti-IL-21 was purchased from eBiosciences (San Diego, CA, USA). After incubating at 4 °C for 30 minutes, samples were washed with PBS and data from the stained cells were acquired using FACSCalibur flow cytometry (BD Biosciences). Flowjo Software (TreeStar, Inc., Ashland, OR) was used for analyzing data.

### Intracellular Staining for IL-21

Isolated PBMCs were stimulated with Phorbol-12-myristate-13-acetate (PMA, 50 ng/ml; Sigma), ionomycin (1 μg/ml; Sangon Biotech, Shanghai, China) for 4 hour, cells were collected for surface marker staining at first step, followed by fixation and permeabilization using an intracellular staining assay (BD, Biosciences) according to manufacturer’s instructions. Cells were then stained with anti-IL-21 for 1 h at room temperature. Data was collected using flow cytometry as described above.

### Measurement of Serum IgG, IgA and IgM

Serum IgG, IgA and IgM were measured by automated Beckman Immage 800 Immunochemistry System (Beckman Coulter) according to the manufacturer’s instructions.

### Statistical Analysis

Data analysis was performed using SPSS 17.0 software (Chicago, IL, USA) and Prism Version 5.04 software (GraphPad, La Jolla, CA). Student *t* test was applied for determining significant difference between two groups. All the data was presented as mean ± standard error of the mean (SEM). Correlations between variables were evaluated by the nonparametric Spearman rank correlation test. P < 0.05 was considered significant different.

## Results

### Serum Levels of IgG Were Decreased in Patients with JORRP

**Figure 1:**
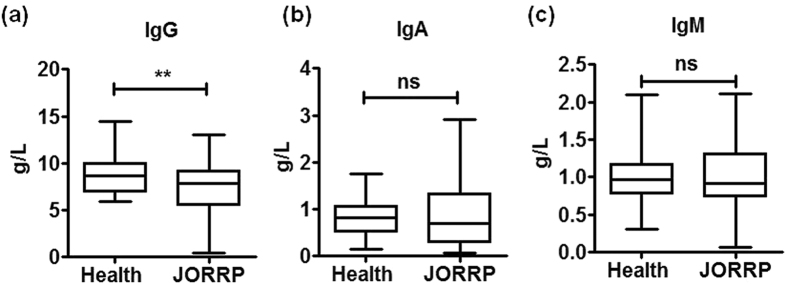
Total serum IgG, IgA, IgM levels in JORRP patients compared with control. Total serum level of IgG (**a**), IgA (**b**) and IgM (**c**) were measured by ELISA in JORRP patients (n = 44) and health controls (n = 37). Data were expressed as the mean ± SEM. ^**^P < 0.01, ns = not significantly different.

Total serum IgG, IgA and IgM levels representing the amplitude of humoral immune response were assessed in JORRP patients. Total serum IgG was significantly decreased in patients with JORRP compared with health controls (mean 8.9 ± 0.4 vs 7.4 ± 0.4 g/L, p = 0.006, Fig. 1a) while IgA and IgM levels were not changed (IgA, mean 0.9 ± 0.1 vs 0.9 ± 0.1 g/L, p = 0.71; IgM, mean 1.0 ± 0.1 vs 1.0 ± 0.1 g/L, p = 0.69. Fig. 1b,c). Collectively, these results indicated that the occurrence of JORRP was associated with decreased serum IgG secretion.

### B Cells Were Expanded With Immature Phenotype in JORRP Patients

**Figure 2:**
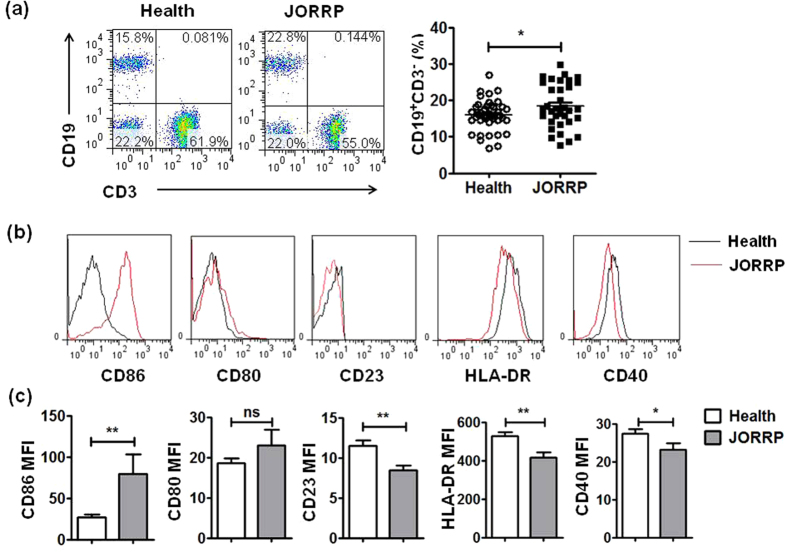
Circulating CD3^−^CD19^+^ B cells were significantly increased in JORRP patients. (**a**) The percentage of CD3^−^CD19^+^ B cells in peripheral blood from JORRP patients (n = 34) and health control (n = 40) were analyzed by flow cytometry. (**b**,**c**) Phenotype analysis of CD3^−^CD19^+^ B cells. Data were expressed as the mean ± SEM. Each dot represents one individual. ^*^P < 0.5, ^**^P < 0.01, ns = not significantly different.

To examine whether decreased serum IgG levels in JORRP patients was a result from a reduction in B cells, we detected the frequency of circulating CD3^−^CD19^+^ B cells in JORRP patients using flow cytometry. Much to our surprise, expanded CD3^−^CD19^+^ B-cell proportion was found in JORRP patients compared with health controls (21.4% ± 1.4 vs 16.1% ± 2.0, p = 0.04, Fig. 2a). Further analysis of surface markers of B-cell maturation and activation in Fig. 2b,c, revealed that CD23, CD40 and HLA-DR expression on B cells were down-regulated in JORRP patients (MFI: CD23, 11.5 ± 0.7 vs 8.4 ± 0.6, p = 0.002; HLA-DR, 526.2 ± 22.0 vs 414.4 ± 28.9, p = 0.003; CD40, 27.4 ± 1.1 vs 23.2 ± 1.7, p = 0.03). In contrast, CD86 expression was significantly up-regulated (MFI: 26.6 ± 3.3 vs 79.1 ± 23.9, p = 0.007) while CD80 expression was not changed in patients with JORRP (MFI: 18.5 ± 1.3 vs 23.0 ± 3.8, p = 0.205).

### Partially Lose of Both Memory B Cells and Plasma Cells in JORRP Patients

**Figure 3:**
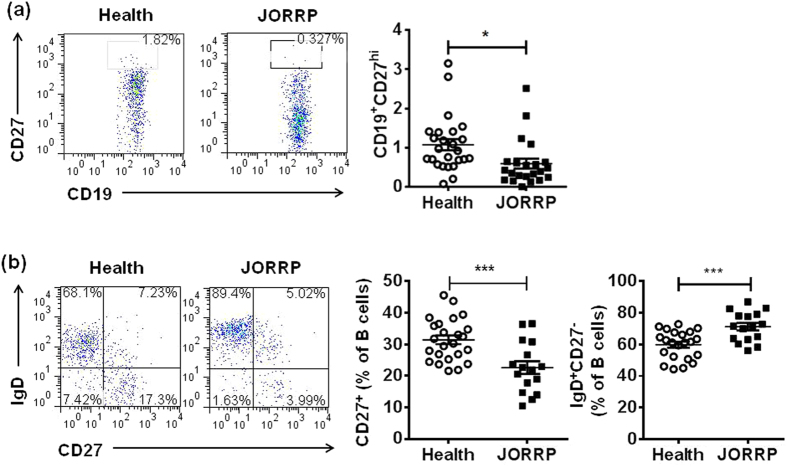
Both plasma cells and memory B cells were significantly decreased in JORRP patients. (**a**) The percentage of CD27^hi^CD19^+^ B cells in peripheral blood from JORRP patients (n = 22) and health control (n = 25) were shown. (**b**) Flow cytometry analysis of IgD^+^CD27^−^ and CD27^+^ of CD3^−^CD19^+^ B cells. Data were expressed as the mean ± SEM. Each dot represents one individual. ^*^P < 0.5, ^***^P < 0.001.

As we observed significant expanded immature B cells in patients with JORRP, we speculated that changes in the B-cell compartment may reduce the ability of IgG-producing B cells. B cells can be divided into CD19^+^CD27^hi^ plasma cells, IgD^+^CD27^−^ naïve B cells, CD27^+^ memory B cells^13^. As shown in Fig. 3, the frequency of both CD19^+^CD27^hi^ plasma cells and CD27^+^ memory B cells were decreased in JORRP individuals compared to health controls (1.1% ± 0.1 vs 0.6% ± 0.1, p = 0.01; 31.4% ± 1.4 vs 22.6% ± 2.0, p = 0.000). We also detected a significantly elevated IgD^+^CD27^−^ naïve B cells in JORRP patients (59.8% ± 1.93 vs 71.2% ± 2.4, p = 0.000). All the data above might indicate that B-cell-related humoral immunity was impaired in JORRP patients.

### The Secretion of IL-21 of Circulating Tfh Cells is Reduced in JORRP Patients

**Figure 4:**
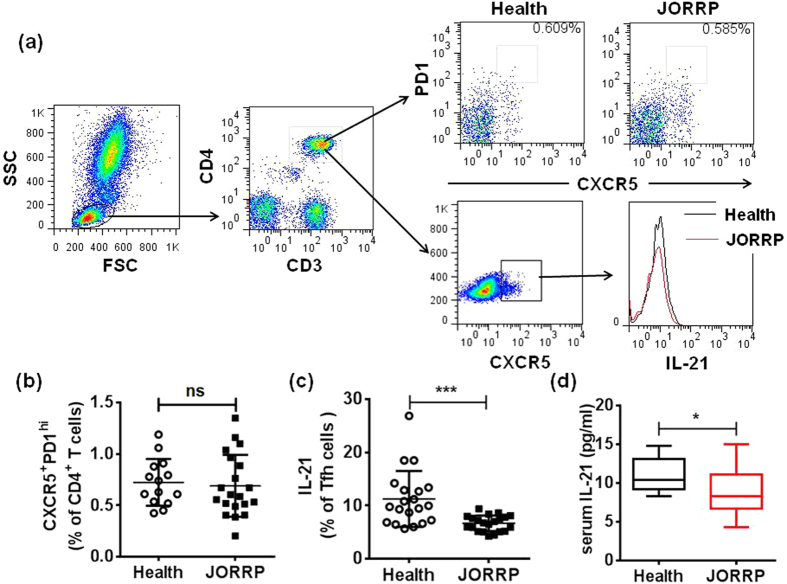
Tfh cells secreted IL-21 was impaired in JORRP patients. (**a**) Gating strategy for CXCR5^+^PD1^hi^ Tfh cells and representative dot plots of the percentage of Tfh cell and IL-21 secretion in JORRP patients (n = 21) and health control (n = 16). (**b**) The percentage of CXCR5^+^PD1^hi^ Tfh cells. (**c**) Intracellular staining of IL-21 in CXCR5^+^CD4^+^ cells in response to PMA/ion stimulation. (**d**) Serum IL-21 level were measured by ELISA. Data were expressed as the mean ± SEM. Each dot represents one individual. ^***^P < 0.001, ns = not significantly different.

**Figure 5:**
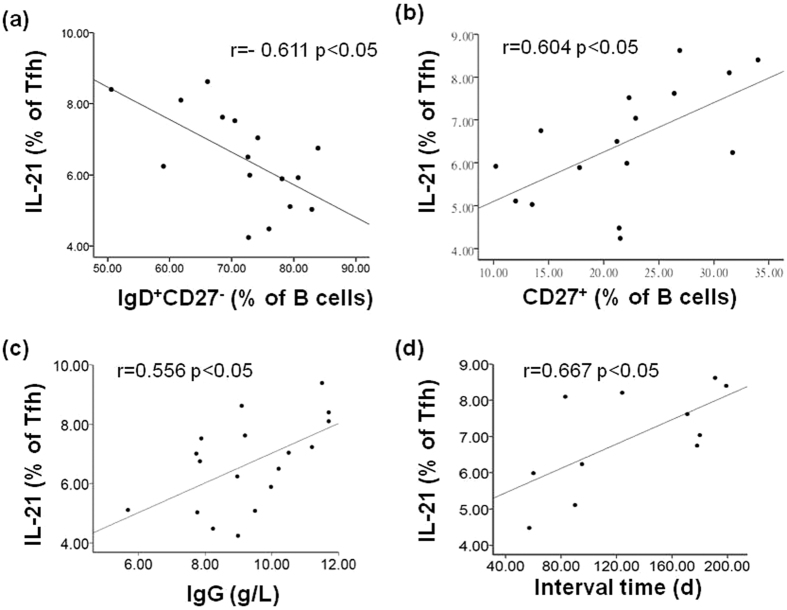
Tfh Cell-derived IL-21 is Correlate to Serum IgG level and Recurrent Frequency in JORRP patients. Correlation of frequency of IL-21 levels secreted by Tfh cells with IgD^+^CD27^−^ (% of B cells) (**a**), CD27^+^ (% of B cells) (**b**), serum IgG level (**c**) and interval time of necessary surgical operation (**d**). Each dot represents one individual.

CXCR5^+^PD-1^hi^ T follicular helper (Tfh) cells producing IL-21 was previously shown to play a pivot role in promoting B cells maturation^10^. In the present study, We use flow cytometry to analyze the frequency of CXCR5^+^PD-1^hi^ Tfh cells in peripheral blood of JORRP patients and found no difference from control group (0.7% ± 0.1 vs 0.7% ± 0.1, p = 0.93, Fig. 4a,b). Neither the expression of ICOS of Tfh cells withheld any significant difference to controls (MFI: 6.6 ± 0.5 vs 7.1 ± 0.9, p = 0.63, Fig. S1). Interestingly, intracellular staining of Tfh-secreted IL-21 reflected that the ability of IL-21 secretion was significantly impaired in Tfh cells from JORRP individuals (11.3% ± 1.2 vs 6.7% ± 0.3, p = 0.000, Fig. 4a,c). Notably, we found a decrease in the serum level of IL-21 in JORRP patients compared with healthy controls (9.0 ± 0.7 vs 11.0 ± 0.5 pg/ml, p = 0.04, Fig. 4d), supplementing our findings in cell compartment. Moreover, CD4^+^ T cells producing IL-21 were found negatively correlated with IgD^+^CD27^−^ cell (r = −0.661, p = 0.01, Fig. 5a) and positively correlated with CD27^+^ B cells (r = 0.604, p = 0.01, Fig. 5b). These results implied that impaired IL-21 secretion in Tfh cells could be the culprit for B cell dysfunction in JORRP patients.

### Tfh Cell-derived IL-21 is Correlate to Serum IgG Level and Recurrent Frequency in Patients with JORRP

Next, we investigated the correlation between the percentage of IL-21 secreted by Tfh cells and serum IgG level or outcome of JORRP. As shown in Fig. 5c, there was a positive correlation between the frequency of IL-21-secreting Tfh cells and serum IgG level (r = 0.556, p = 0.01), but no correlation was found with serum IgA or IgM level (IgA, r = 0.063, p = 0.81; IgM, r = 0.300, p = 0.226, Fig. S2). Moreover, IL-21 levels secreted by Tfh cells predicted longer interval time of recurrent occurrence in JORRP individuals (r = 0.667, p = 0.02, Fig. 5d) but not the number of surgical interventions (r = 0.300, p = 0.278, Fig. S3). These results suggest that decreased Tfh-secreting IL-21 might be associated with poor JORRP outcome in clinic.

## Discussion

Both cellular and humoral immunity play key roles in protecting host against virus infection^14^,^15^,^16^. While dysfunction of cellular immune responses against HPV type 6 or 11 virus infection are described in various studies^6^,^7^, whether humoral immunity involves in the progression of JORRP remains unknown. In the present study, we examined B-cell related humoral response and observed a significant expanded immature B cells in patients diagnosed with JORRP compared to healthy controls. Moreover, changes in the B-cell compartment with decreased plasma and memory B cells reduced the protective IgG production in JORRP individuals. Furthermore, we demonstrated that reduced IL-21 secretion by Tfh cells prevented B cells from maturation and was associated with less serum IgG production or poor prognosis of JORRP regarding the interval time of disease recurrence.

In previous studies, ELISA analysis of eighteen healthy female volunteers aged 19–31 years immunized with HPV vaccine showed that HPV vaccine could elicit circulating IgG- and IgA- secreting cells^17^. By detecting HPV-specific IgG and IgA in adolescent girls after HPV vaccination, Scherpenisse *et al*. demonstrated a long-term virus-specific IgG and IgA concentration in serum^18^. In our study, serum total IgG was found decreased in JORRP patients while serum IgA or IgM remained unchanged compared to healthy controls. We showed that only total IgG but not IgA was decreased in JORRP patients. Our results indicated a potential use of HPV vaccine to elicit IgG-producing B cells to neutralize HPV virus in the treatment of JORRP. However, whether the secretion of HPV 6/11 virus specific IgG impaired in JORRP patients was not addressed in our current issue and still need further investigation.

We first observed an expansion of circulating CD3^−^CD19^+^ B cells in peripheral blood of JORRP patients, however, the frequency of plasma cells and memory B cells were greatly decreased. Our findings suggested that although the increased percentage of B cells was observed in JORRP patients, the maturation of B cells are limited. This may explain why serum IgG was significantly decreased in JORRP patients in the context of expanded CD19^+^ B cells. Further study was needed to explore whether germinal center (GC) formation was also affected in papillomatosis tissue.

IL-21, mainly secreted by Tfh cells, is the major factor that drives B-cell differentiation and plasma cell responses^11^,^19^,^20^,^21^. Dysfunction of CXCR5^+^PD1^hi^ Tfh cells was associated with inflammatory and autoimmune diseases, as well as cancer development^22^,^23^,^24^. Intracellular staining of IL-21 in CXCR5^+^CD4^+^ cells showed that circulating Tfh cells from JORRP patients exhibited a deficiency IL-21 production compared with those from healthy controls in our study. Katsutoshi *et al*. reported a critical role of IL-21 in IgG production^25^, which IL-21 induced CD40L-stimulated human naïve B cells to undergo IgG class switch^26^. In accordance with their results, we demonstrated that IL-21 produced by Tfh cells was correlated to serum IgG level, which implied an important role in regulating humoral immune response against HPV-6/11 infection in JORRP patients. A requirement of IL-21 in promoting Tfh cell differentiation has been well documented as a reduced number of Tfh cells was observed in IL-21^−/−^ mice^27^,^28^,^29^. In another study, IL-21^−/−^ mice showed a normal Tfh cells formation but a faster turnover, suggesting that IL-21 is required for Tfh cells maintenance instead of fostering Tfh cell formation^10^. Interestingly, in our study, although IL-21 production was impaired, the frequency of CXCR5^+^PD^hi^ Tfh cells was not decreased in peripheral blood of JORRP patients. Although circulating Tfh cells share similar phenotype and function with Tfh cells on the anatomical location^30^, whether the percentage of tissue-infiltrated Tfh cells were decreased in JORRP patients remains unknown.

Our study have limitations in that the correlation between humoral immunity or Tfh cells-derived IL-21 and HPV virus tilters was not addressed as we failed to detect HPV-6/11 virus in the peripheral blood since HPVs infect locally and do not cause viremia. Therefore HPV typing was not performed in our study and whether humoral immune response was associated with virus type or virus load remains unknown. Moreover, currently there was not a definite criterion of the severity of JORRP, our study only demonstrated that enhanced IL-21 secretion by Tfh cells might prolong the recurrence interval. Whether circulating Tfh-secreting IL-21 could be a reliable prognostic marker of JORRP still needs further exploration.

In conclusion, our present study demonstrated that reduced secretion of IL-21 by Tfh cells hampered the maturation of B cells, which led to a decrease in IgG-producing B cells. It is likely an upstream factor for poorer prognosis in JORRP patients. Our findings highlight the role of Tfh cell-mediated humoral immune response in JORRP patients and might provide a potential therapeutic target for the treatment of JORRP.

## Additional Information

**How to cite this article**: Wu, X. *et al*. Impaired T Cell-dependent Humoral Immune Response Associated with Juvenile-onset Recurrent Respiratory Papillomatosis Progression. *Sci. Rep.* **6**, 36378; doi: 10.1038/srep36378 (2016).

**Publisher’s note:** Springer Nature remains neutral with regard to jurisdictional claims in published maps and institutional affiliations.

## Supplementary Material

Supplementary Information

## Figures and Tables

**Table 1:** Demographic and Clinical Characteristics.

Variables	Parameters
Healthy Controls (n = 40)	
Gender (Male/Female)	26/14
Age (month)	47.2 ± 4.0
JORRP (n = 44)	
Gender (Male/Female)	25/19
Age (month)	46.5 ± 4.7
Active of RRP	44 (100.0)
No. of surgeries	
N< = 3, No. (%)	25 (56.8)
N > 3, No. (%)	19 (43.2)
Age at onset	
<3 years, No. (%)163	35 (79.5)
>3 years, No. (%)	9 (20.5)
Disease Duration	
<1 year, No. (%)	21 (47.7)
>1 year, No. (%)	23 (52.3)
