# Supplementary material for: Impaired T Cell-dependent Humoral Immune Response Associated with Juvenile-onset Recurrent Respiratory Papillomatosis Progression
Source: Sci Rep. 2016 Nov 8;6:36378. doi: 10.1038/srep36378 (PMC5099571; doi:10.1038/srep36378)
Supplement: Supplementary Information [file srep36378-s1.doc]

**Impaired T Cell-dependent Humoral Immune Response Associated with Juvenile-onset Recurrent Respiratory Papillomatosis Progression**

Xunyao Wu^1,2,*^, Guoliang Wang^1,2,*^, Xi Chen^1,2^,, Jie Zhang^4^,, Jing Zhao^4^,, Jun Wang^5^,, Yang Xiao^5^,, Jun Tai^4^,, Shengcai Wang^4^,, Guixiang Wang^4^,, Hua Wang^4^,, Lina Bai^5^, Jingang Gui^1,2,#^, Xin Ni^1,3,4,#^

*These authors contributed equally to this work.

1. Key Laboratory of Major Diseases in Children, Ministry of Education, Beijing Children’s Hospital, Capital Medical University, Beijing, China

2. Laboratory of Immunology, Beijing Pediatric Research Institute, Beijing Children’s Hospital, Capital Medical University, Beijing, China

3. Beijing Key Laboratory for Pediatric Diseases of Otolaryngology, Head and Neck Surgery, Beijing Pediatric Research Institute, Beijing Children's Hospital, Capital Medical University, Beijing, China.

4. Department of Otolaryngology, Head and Neck Surgery, Beijing Children's Hospital, Capital Medical University, Beijing.

5. Department of Otolaryngology Head and Neck Surgery, Beijing Tongren Hospital, Capital Medical University, Beijing, China.

# Correspondence should be addressed to:

Xin Ni (MD. Ph.D.)

Email: Nixin@bch.com.cn

Department of Otolaryngology, Head and Neck Surgery, Beijing Children's Hospital, Capital Medical University, Beijing, 100045, China

and

Jingang Gui (Ph.D.)

Email: [guijingang@bch.com.cn](mailto:guijingang@bch.com.cn)

Laboratory of Immunology, Beijing Pediatric Research Institute, Beijing Children’s Hospital, Beijing, 100045, China

**Authors:**

Xunyao Wu, PhD, email: [bchyaoyao@163.com](mailto:bchyaoyao@163.com)

Guoliang Wang, PhD, email: [wgl163@126.com](mailto:wgl163@126.com)

Xi Chen, MD, email: [cx1000@sina.com](mailto:cx1000@sina.com)

Jie Zhang, email: [stzhangj@263.net](mailto:stzhangj@263.net)

Jing Zhao, email: [zhaojing307@163.com](mailto:zhaojing307@163.com)

Jun Wang, email: [wmzi2002@163.com](mailto:wmzi2002@163.com)

Yang Xiao, email: [18910137727@163.com](mailto:18910137727@163.com)

Jun Tai, PhD, email: [trenttj@163.com](mailto:trenttj@163.com)

Shengcai Wang, MD, email: [wsc820329@163.com](mailto:wsc820329@163.com)

Guixiang Wang, email: [wgx_ent@sina.com](mailto:wgx_ent@sina.com)

Hua Wang, email: [wanghua19720703@126.com](mailto:wanghua19720703@126.com)

Lina Bai, email: [bailina0410@sina.com](mailto:bailina0410@sina.com)

Jingang Gui, PhD, email: [guijingang@bch.com.cn](mailto:guijingang@bch.com.cn)

Xin Ni, PhD, email: [Nixin@bch.com.cn](mailto:Nixin@bch.com.cn)


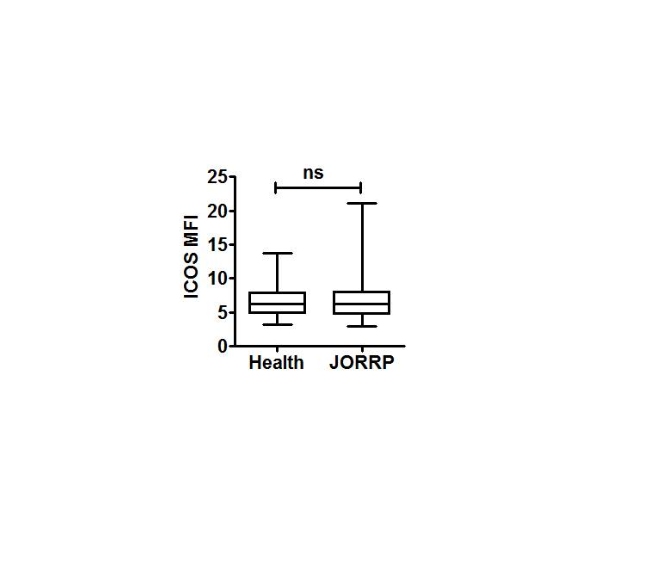


**Figure S1. ICOS expression on Tfh cells were not changed between JORRP patients and health controls.**

Flow cytometry analysis of mean fluorescence intensity (MFI) of ICOS on the surface of CD4^+^CXCR5^+^ cells in JORRP patients and health control, ns=not significantly different.


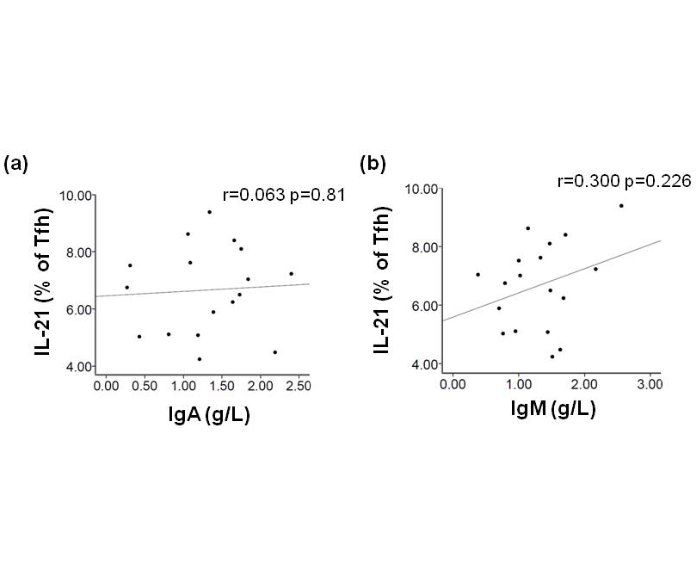


**Figure S2. Correlation of Tfh cell-derived IL-21 with serum IgA level and IgM level in JORRP patients.**

Correlation of frequency of IL-21 levels secreted by Tfh cells with serum IgA level (a) and serum IgM level (b). Each dot represents one individual.


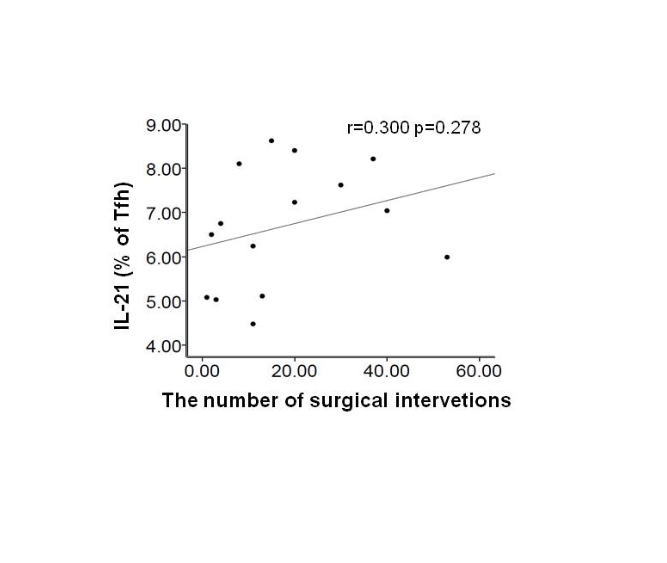


**Figure S3. Correlation of Tfh cell-derived IL-21 with the number of surgical interventions in JORRP patients.**

Correlation of frequency of IL-21 levels secreted by Tfh cells with the number of surgical interventions. Each dot represents one individual.
